# Cytochrome P450 Enzymes as Key Drivers of Alkaloid Chemical Diversification in Plants

**DOI:** 10.3389/fpls.2021.682181

**Published:** 2021-07-02

**Authors:** Trinh-Don Nguyen, Thu-Thuy T. Dang

**Affiliations:** Department of Chemistry, Irving K. Barber Faculty of Science, University of British Columbia, Kelowna, BC, Canada

**Keywords:** alkaloid, catalysis, scaffold, diversification, oxidation, medicinal plants, P450

## Abstract

Plants produce more than 20,000 nitrogen-containing heterocyclic metabolites called alkaloids. These chemicals serve numerous eco-physiological functions in the plants as well as medicines and psychedelic drugs for human for thousands of years, with the anti-cancer agent vinblastine and the painkiller morphine as the best-known examples. Cytochrome P450 monooxygenases (P450s) play a key role in generating the structural variety that underlies this functional diversity of alkaloids. Most alkaloid molecules are heavily oxygenated thanks to P450 enzymes’ activities. Moreover, the formation and re-arrangement of alkaloid scaffolds such as ring formation, expansion, and breakage that contribute to their structural diversity and bioactivity are mainly catalyzed by P450s. The fast-expanding genomics and transcriptomics databases of plants have accelerated the investigation of alkaloid metabolism and many players behind the complexity and uniqueness of alkaloid biosynthetic pathways. Here we discuss recent discoveries of P450s involved in the chemical diversification of alkaloids and how these inform our approaches in understanding plant evolution and producing plant-derived drugs.

## Introduction

### Alkaloids – A Functionally and Structurally Diverse Natural Product Class With Unique Underlying Biosynthesis

Chemical diversity is the key to success for the sessile lifestyle that plants have evolved to adapt. Over hundreds of millions of years, land plants have accumulated a formidable capacity to biosynthesize numerous small molecules, often referred to as natural products or specialized metabolites, that help them thrive in specific environmental niches. Among plant natural products, alkaloids constitute arguably the most intriguing class with thousands-of-years interconnection with human history. Alkaloids have long been used and abused for their potent therapeutic properties and notorious toxic and psychedelic effects with significant geopolitical impacts, as seen in the Anglo-Chinese opium wars of the 19th century and the ongoing war on drugs (Kutchan et al., 2015). Moreover, alkaloid diversity has attracted much attention from chemists, biologists, and pharmacologists alike for its unique structural diversification pathways. Instead of sharing the same biosynthetic routes as observed in terpenoid metabolism, the common nitrogen-containing heterocyclic structure of more than 20,000 known alkaloids can be generated by various Mannich-like condensation of amino acids-derived iminiums (Lichman, 2021). The resulted alkaloid scaffolds are then decorated and modified extensively to form a wide range of structures, ranging from the poisonous coniine with a simple eight-carbon and one-nitrogen skeleton naturally occurring in hemlock (*Conium maculatum*) to the complex anti-tumor drug vinblastine with a dimeric 45-carbon and four-nitrogen scaffold found in Madagascar periwinkle (*Catharanthus roseus*; Ziegler and Facchini, 2008; O’Connor, 2010).

For example, in monoterpenoid indole alkaloid (MIA) biosynthesis, the amine moiety from tryptamine, a derivative of the amino acid tryptophan, is condensed with the aldehyde moiety from secologanin, a member of the non-canonical monoterpenoid group called iridoids, to yield strictosidine. From this central precursor, different multiple-step pathways are catalyzed by scaffolding and tailoring enzymes such as cytochrome P450 monooxygenases (P450), 2-oxoglutarate-dependent dioxygenases, methyltransferases, dehydrogenases, acetyltransferases, and glycosyltransferases. This leads to more than 2,000 MIA structures mostly found in the dogbane family (Apocynaceae), with vinblastine as the best-known example. Other illustrating examples are found in the biosynthesis of benzylisoquinoline alkaloids (BIAs) which starts with the condensation of the amine moiety of dopamine and the aldehyde group of 4-hydroxyphenylacetaldehyde, both derived from the amino acid tyrosine. The resulted (*S*)-norcoclaurine goes through series of structural changes including oxidation, reduction, methylation, acetylation, and decarboxylation to yield approximately 2,500 BIA structures such as the well-known narcotic painkiller morphine in opium poppy (*Papaver somniferum*; Ziegler and Facchini, 2008; O’Connor, 2010; Dastmalchi et al., 2018; Desgagné-Penix, 2021).

### Plant P450s and Chemical Diversity in Plants

Dubbed “nature’s most versatile biological catalyst,” P450s display incredible adaptability in all domains of life and even in certain viruses (Coon, 2005). Starting as a component of the ancient cell’s biochemical response to a world filled with the newly-abundant and poisonous oxygen gas (Wickramasinghe and Villee, 1975), P450s’ ability to scissor atmospheric dioxygen at physiological temperatures has turned them into a reservoir of catalysts whose members have been recruited over and again in various metabolic pathways. The structure of a typical P450 consists of a central haem iron tethered by the thiolate group of a cysteine residue. This arrangement allows the formation of the highly reactive Fe^IV^-oxo species, which abstracts hydrogen from the substrate’s chemically inert C–H bond and can kick start a cascade of structural diversification and functionalization with high selectivity, a catalytic feat enviable to chemists (Lewis et al., 2011). Not only from its bond with carbon, hydrogen can also be abstracted by P450 enzymes from bonds with nitrogen, oxygen, and sulfur to allow oxidation and a range of other reactions such as epoxidation, sulfoxidation, dehydrogenation, aryl–aryl coupling and dehalogenation (Coon, 2005; Guengerich and Munro, 2013; Lamb and Waterman, 2013). From these initial chemical changes, carbon skeleton re-arrangements can further expand the chemical space (Tang et al., 2017; Zhang and Li, 2017).

P450 diversity reflects the evolution of metabolism and adaptation in living organisms, especially plants, fungi, and bacteria, whose chemical diversity is extraordinary. In plants, hundreds of thousands of P450s have been identified and grouped in 277 families of sequences sharing 40% or higher identity; of these, more than 16,000 have been named (Nelson, 2018). P450s control many metabolic steps and pathways of plant primary metabolites such as the growth regulators gibberellins, brassinosteroids, and abscisic acid (Helliwell et al., 2001; Turk et al., 2003; Kitahata et al., 2005). P450 enzymes also play a crucial role in plants’ eco-physiological adaptation as they catalyze the production of defensive compounds and allelochemicals, among other specialized metabolites. Indeed, the vast majority of plant natural products are oxygenated, and, as most oxidations of chemicals in the living world are catalyzed by P450s, these proteins constitute the largest superfamily of enzymes underlying the diversification of plant natural products (Nelson and Werck-Reichhart, 2011; Hamberger and Bak, 2013; Guengerich, 2018).

Increasing genomic and transcriptomic data in recent years have facilitated the characterization of hundreds of P450s involved in plant specialized metabolism. Here we review the roles of P450s in the structural diversification of plant alkaloids with select recently-elucidated examples being discussed in accordance with the chemical modifications they catalyze (e.g., oxygenation, scaffold re-arrangement, etc.). The metabolism of major alkaloid groups (i.e., BIA, MIA, etc.) which involve these P450s are summarized in the figures with more details available in several excellent reviews published in the past years (Hagel and Facchini, 2013; Larsson and Ronsted, 2013; Thamm et al., 2016; Frick et al., 2017; Dastmalchi et al., 2018; Polturak and Aharoni, 2018; Zenkner et al., 2019; Desgagné-Penix, 2021; Lichman, 2021). Given the unique chemical diversification of alkaloids, insights into the power of P450-based biocatalysts offer essential lessons for exploring unknown pathways as well as generating new-to-nature chemical diversity with tremendous potential applications.

## Oxygenation as a Starting Point for Chemical Diversification

The most common reaction catalyzed by P450s is the addition of an oxygen atom into the substrate molecule in the form of a hydroxyl or an epoxide group. This has particularly relevant implications in biotechnologies and pharmaceutical industries as the oxidation of a single C–H bond functionalizes many compounds or makes them more biologically active. For instance, the stereo- and regio-selective oxidations of camptothecin and compactin lead to their more potent hydroxylated forms hydroxycamptothecin (anti-cancer) and pravastatin (lipid-lowering), respectively (Kingsbury et al., 1991; Watanabe et al., 1995), and the underlying oxidases can address industrial-scale drug production issues (Di Nardo and Gilardi, 2020). Furthermore, these simple oxygenations prompt a whole host of additional chemical decorations on the molecules, such as methylation, acetylation, glycosylation, and structural re-arrangements in many pathways.

**Figure 1:**
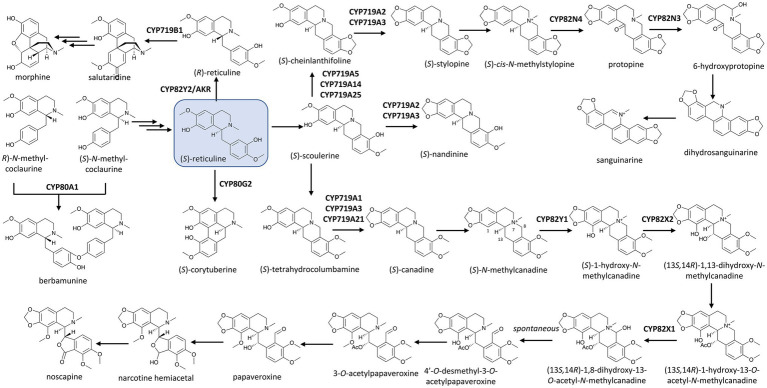
P450s in the biosynthetic network of benzylisoquinoline alkaloids (BIAs) in the Ranunculales with (*S*)-reticuline (boxed) as the central precursor. Multiple and single arrows indicate multiple- and single-step pathways, respectively. Enzymes indicated here are discussed in the text.

The recently-elucidated BIA biosynthetic pathways feature several P450s that catalyze such oxygenations. In noscapine biosynthesis in opium poppy (*P. somniferum*), three members of the CYP82 family add single hydroxyl groups to the *N*-methylcanadine scaffold at three different positions with different chemical fates in the end product, noscapine. The first committed step of the pathway was catalyzed by CYP82Y1, hydroxylating (*S*)-*N*-methylcanadine at C1 position (Dang and Facchini, 2014b). The second and third P450s, CYP82X2 and CYP82X1, hydroxylates at C13 and C8 positions, respectively (Dang et al., 2015; Figure 1). While the 1-hydroxyl group undergoes a methylation reaction later in the pathway, the 8-hydroxyl group constitutes an unstable structure with the adjacent quaternary ammonium group and is spontaneously converted to an aldehyde group by breaking the C8–N7 bond. This newly-formed C8 aldehyde group then forms a hemiacetal ring with the 13-hydroxyl group. Intriguingly, before forming the hemiacetal structure with the 8-hydroxyl group, the 13-hydroxyl group undergoes acetylation and subsequent deacetylation before and after 8-hydroxylation by CYP82X1. As CYP82X1 and CYP82X2 do not accept each other’s substrates, this acetylation seems to protect the oxygenated moiety at C13 and allow both 13- and 8-hydroxylations to occur, albeit in strict order (Dang et al., 2015).

Other members of the CYP82 family have also been found to be responsible for ring hydroxylations of BIAs. In the biosynthesis of the anti-microbial BIA sanguinarine, CYP82N4 catalyzes the hydroxylation at C14 of (*S*)-*cis*-*N*-methylstylopine, breaking the C14–N7 bond to yield protopine. Protopine is in turn hydroxylated by CYP82N3 in opium poppy (Beaudoin and Facchini, 2013) and by CYP82N2 in California poppy (*Eschscholzia californica*; Takemura et al., 2013) to 6-hydroxyprotopine, which is spontaneously converted to dihydrosnaguinarine, illustrating how hydroxylations by P450s can lead to further structural re-arrangement (Figure 1).

**Figure 2:**
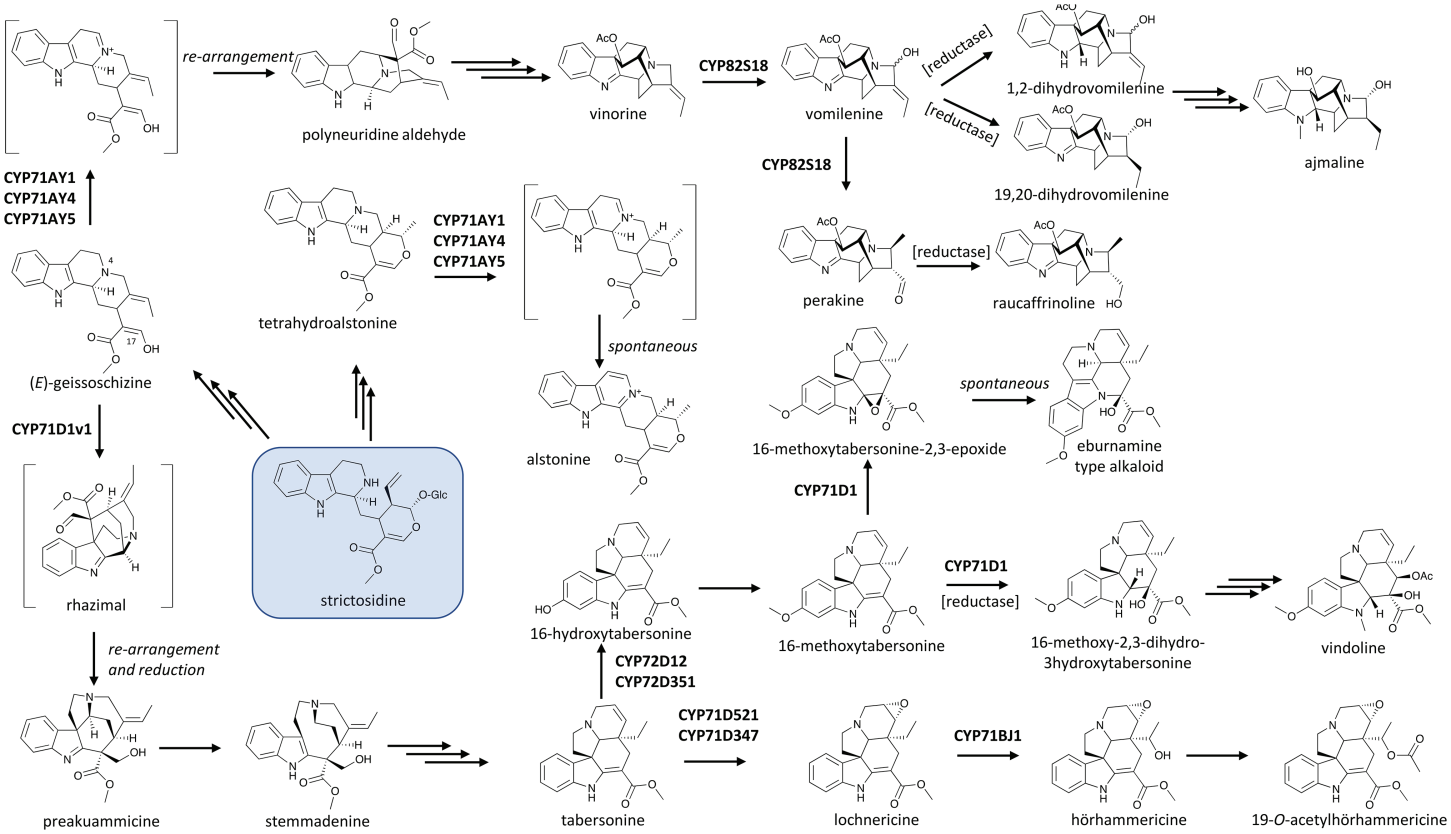
P450s in the biosynthetic network of monoterpenoid indole alkaloids (MIAs) in the Apocynaceae with strictosidine (boxed) as the central precursor. Multiple and single arrows indicate multiple- and single-step pathways, respectively. Enzymes indicated here are discussed in the text. V19H: (+)-vincadifformine 19-hydroxylase, a close homologue of CYP71D1.

CYP82S18, a unique P450 involved in MIA metabolism in Indian snakeroot (*Rauwolfia serpentina*), catalyzes not only the ring hydroxylation of vinorine to form vomilenine, but also the non-oxidative isomerization of this product to perakine (Figure 2; Dang et al., 2017). Although enzymes are not required for the conversion of vomilenine to perakine, it needs extreme chemical catalysis conditions (Taylor et al., 1962), and biochemical studies showed that plant enzymes facilitate the isomerization (Sun et al., 2008; Dang et al., 2017). It remains unclear how CYP82S18 catalyzes this non-oxidative structural change; however, data suggest that keeping the product vomilenine in the active site after the hydroxylation of vinorine is essential, and a series of re-arrangements *via* ring opening and Michael addition could be facilitated by this active site (Dang et al., 2017). Indian snakeroot’s CYP82S18 could be considered as an example of a “moonlighting” P450 that can catalyze different types of structural transformation on the substrate, although it does not use different active sites as seen the “moonlighting” terpene synthase/oxidase CYP170A1 in *Streptomyces coelicolor* (Zhao et al., 2009). More importantly, this unique catalytic capacity of CYP82S18 underlies the divergence of MIA metabolism in Indian snakeroot as vomilenine is the central intermediate leading to a series of MIAs, including the antiarrhythmic drug ajmaline while the bifurcated perakine branch leads to raucaffrinoline. The 21-hydroxyl group of vomilenine resulted from the CYP82S18’s hydroxylase activity also allows subsequent glycosylation in the end products of these divergent pathways (Figure 2).

Examples of P450s from other families involved in alkaloid hydroxylation can be widely found in the metabolism of MIAs in the alkaloids-rich dogbane family (Apocynaceae). As early as in the 1990s, the P450-based 16-hydroxylation of tabersonine, a precursor of many MIAs, was identified in Madagascar periwinkle (*C. roseus*; St-Pierre and De Luca, 1995; Schröder et al., 1999). This P450, CYP71D12, yields 16-hydroxytabersonine, the branching precursor leading from tabersonine to vindoline, which together with catharanthine forms the anti-cancer drug vinblastine (Figure 2). More recently, a homologue sharing 82% amino acid identity to CYP71D12 and CYP71D351 was found to be another tabersonine 16-hydroxylase. CYP71D351, in contrast to CYP71D12, is expressed in better correlation with other vindoline biosynthetic genes. This suggests that it plays a major role in the biosynthesis of vindoline, which is tightly controlled in an organ-dependent manner and accumulates mostly in leaves of Madagascar periwinkle (Besseau et al., 2013). The 16-hydroxyl group of tabersonine allows a methyl group to be transferred onto the molecule, and both the resulted 16-methoxytabersonine and tabersonine can be further oxidized by another CYP71D subfamily member, CYP71D1, to yield the corresponding 2,3-epoxides (Kellner et al., 2015; Qu et al., 2015; Edge et al., 2018). Intriguingly, yeast feeding assay suggests CYP71D1 converts 16-methoxytabersonine to its 2,3-epoxide, which subsequently undergoes re-arrangement to an eburnamine scaffold similar to that of the anti-hypertension drug vincamine (Kellner et al., 2015; Figure 2). Reports by Qu et al. (2015) and Edge et al. (2018) as well as the early work by Wenkert and Wickberg (1965) suggest that such a re-arrangement of the 2,3-epoxide intermediate is induced by the acidification of the yeast culture or extraction process. Furthermore, the concerted activities of CYP71D1 and tabersonine-3-reductase were reported to reduce the C2–C3 double bond and hydroxylate C3 of tabersonine and 16-methoxytabersonine. It is the products of these oxidoreduction catalyzes, not the epoxides, that serve as precursors to vindorosine and vindoline, respectively (Qu et al., 2015; Edge et al., 2018; Figure 2). These transformations, underlined by enzymatic activity or otherwise, highlight the frequent occurrence and potential applications of oxidation-induced re-arrangements in MIAs.

The divergence of tabersonine metabolism involves other members of the CYP71 family. CYP71BJ1 was implicated in the biosynthesis of 19-*O*-acetylhörhammericine as it hydroxylates the tabersonine scaffold at C19 and thus allows the acetylation at this position in the final product (Figure 2; Giddings et al., 2011). More recently, Carqueijeiro et al. (2018) found two CYP71D homologues, CYP71D347 and CYP71D521, which catalyze the same 6,7-epoxidation of tabersonine to lochnericine. As both of these epoxidases exhibit strict substrate specificity towards tabersonine while the 19-hydroxylase CYP71BJ1 can accept both tabersonine and lochnericine, the 6,7-epoxidation appears to be the first step in the pathway leading to 19-*O*-acetylhörhammericine from tabersonine (Figure 2). Intriguingly, the substrate spectrum of CYP71BJ1 may not extend to other aspidorsperma MIA enantiomers. In addition to tabersonine and catharanthine, the stemmadenine pathway gives rise to (+)-vincadifformine. This compound is hydroxylated at C19 position by (+)-vincadifformine 19-hydroxylase. Although this reaction is almost identical to the 19-hydroxylations of tabersonine and lochnericine (tabersonine-6,7-epoxide) catalyzed by CYP71BJ1, (+)-vincadifformine 19-hydroxylase shares a higher sequence identity (about 80%) to CYP71D1 compared to its identity to CYP71BJ1 (37%; Williams et al., 2019). The hydroxylation of (+)-vincadifformine and subsequent acetylation leading to (+)-echitovenine, parallel with the 19-*O*-acetylhörhammericine route, underscores enzymatic stereo-selectivity as a critical feature in defining similar yet distinct pathways in MIA diversification (Figure 2).

**Figure 3:**
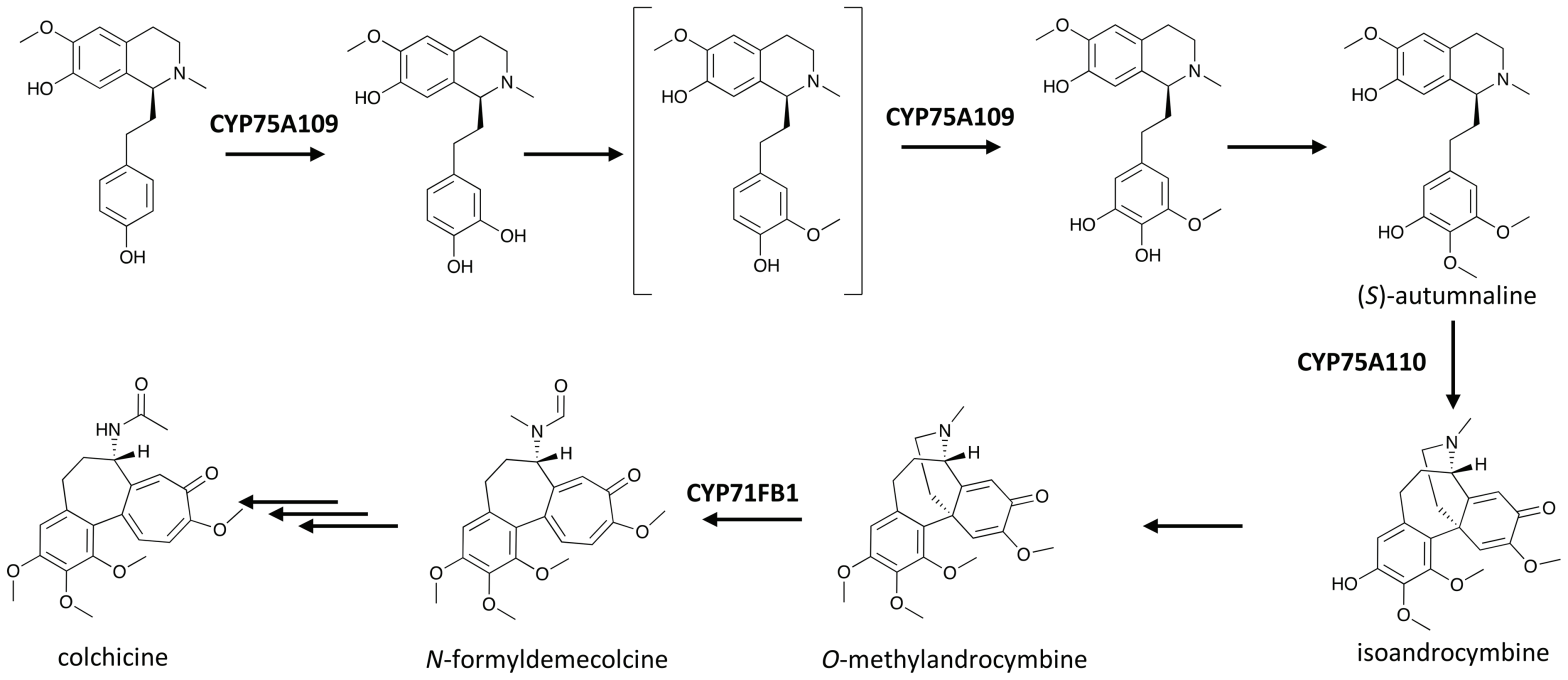
P450s in the recent near elucidation of colchicine biosynthesis in the Colchicaceae. Multiple and single arrows indicate multiple- and single-step pathways, respectively. Enzymes indicated here are discussed in the text.

A member of the CYP75A subfamily responsible for two hydroxylations of the same pathway was featured in the recent near-complete elucidation of colchicine biosynthesis (Nett et al., 2020). Colchicine from *Colchicum* and *Gloriosa* species has long been used to treat inflammations, including gout and Behçet’s disease (Barnes, 2006). It has been hypothesized that the biosynthesis of colchicine involves the condensation of 4-hydroxydihydrocinnamaldehyde and dopamine, derived from l-phenylalanine and l-tyrosine, respectively, to yield the 1-phenethylisoquinoline structure, which is then methylated, hydroxylated, and rearranged in several steps to form the tropolone ring in colchicine. Nett et al. (2020) discovered that CYP75A109 catalyzes not only one but possibly two hydroxylations at two *meta* positions on ring A of the 1-phenethylisoquinoline scaffold (Figure 3), and both of the resulting hydroxyl groups are later methylated in the pathway.

## Oxidative Scaffold Formation

Cyclization reactions that give rise to complex polycyclic scaffolds are hallmarks of alkaloid biosynthetic pathways. While synthetic efforts since the dawn of organic synthesis have demonstrated how different alkaloids can be chemically synthesized from a common scaffold, the enzymes, mostly P450s, that control the regio- and stereo-specific re-arrangement and chemical diversification of the central intermediate found in biological systems have largely remained cryptic until recently. In MIA biosynthesis, a key scaffold forming step is catalyzed by sarpagan bridge enzyme (SBE) and transforms the skeletal scaffold of the central MIA intermediate strictosidine into sarpagan, ajmalan and alstophyllan alkaloid classes (Namjoshi and Cook, 2016), including the class Ia antiarrhythmic agent ajmaline and the anti-cancer compound koumine (Hashimoto et al., 1986; Zhang et al., 2015). More than 20 years after SBE activity was first detected in plants (Schmidt and Stöckigt, 1995), three P450 homologues in *R. serpentina* (CYP71AY4), *Gelsimium sempervirens* (CYP71AY5) and *C. roseus* (CYP71AY1) were found to be responsible for the formation of this scaffold (Dang et al., 2018). The SBE-catalyzed oxidation does not involve oxygenation but the formation of a Schiff base at N4 position of geissoschizine, a strictosidine derivative. This allows a skeletal re-arrangement to form polyneuridine aldehyde, the entry intermediate to the ajmalan-type and alstophyllan-type alkaloids (Figure 2). Intriguingly, when tested with a range of other MIAs, these SBEs turnover tetrahydroalstonine, a heteroyimbine alkaloid, to alstonine *via* a similar iminium intermediate. Guided by related yet structurally distinct substrates, the SBEs can catalyze either cyclization to form a sarpagan bridge or aromatization of the alkaloid scaffold and thus redirect and diversify the pathway at critical points (Dang et al., 2018). This illustrates how P450s’ catalytic and substrate promiscuity, along with the inherent reactivity of these alkaloid substrates, can create a suite of structurally diverse chemical products in many pathways.

Besides acting alone, P450 enzymes can function in combination with other enzymes to open gateways into rich families of natural products. It is not an exception that concerted action of enzymes from two groups commonly involved in plant natural product metabolism, the P450s and the reductases, produces unexpected re-arrangements leading to different scaffolds. For instance, the CYP71/reductase module was observed in strychnos, sarpagan, ajmalane and *β*-carboline in MIA biosynthesis. Specifically, geissoschizine synthase (an alcohol dehydrogenase), geissoschizine oxidase (a P450), and two other reductases from *C. roseus*, when assayed simultaneously, catalyzed a series of tandem reactions that lead to the remarkable re-arrangement of the tetrahydro-*β-*carboline strictosidine substrate into the corynanthean, strychnos, iboga, and aspidosperma scaffolds (Benayad et al., 2016; Tatsis et al., 2017; Qu et al., 2018a). The alcohol group at C17 of geissoschizine is oxidized to an aldehyde by CYP71D1v1, triggering a cascade of skeletal changes and a reduction catalyzed by two conceptive reductases (Tatsis et al., 2017; Qu et al., 2018a,b; Figure 2). The resulting preakuammicine is then either spontaneously transformed to akuammicine or reduced to stemmadenine, the precursor to tabersonine and catharanthine (Caputi et al., 2018).

The dual catalytic function of vinorine hydroxylase, CYP82S18, in the ajmaline biosynthetic pathway mentioned earlier is also driven by the presence of different downstream aldo/keto reductases (AKRs). Various combinations of these reductases with CYP82S18 diverge the vomilenine pool to tissue-specific metabolic routes with different end-products, including ajmaline, raucaffrinoline, raucaffricine, rauglucine, and 21-hydroxysarpagane glucoside (Figure 2; Dang et al., 2017). Intriguingly, an unusual P450, CYP82Y2, was identified to be a domain of a fused protein that also includes an AKR domain, and this fused AKR/P450 catalyzes the isomerization of (*S*)-reticuline to its (*R*)-epimer, a precursor of morphine biosynthesis in opium poppy (Farrow et al., 2015; Galanie et al., 2015; Winzer et al., 2015; Figure 1). What is more unusual is that in contrast with other CYP82 enzymes, which usually catalyze ring hydroxylation of BIAs (Dang and Facchini, 2014b; Dang et al., 2015), CYP82Y2 in reticuline isomerization removes hydrogen from (*S*)-reticuline to yield 1,2-dehydroreticuline. The resulted double bond is in turn reduced by the AKR domain of the AKR/CYP82Y2 fusion to produce (*R*)-reticuline. Similar fusion proteins made up of a CYP82Y2-like portion and an AKR domain were also found in dwarf breadseed poppy (*P. setigerum*) and Persian poppy (*P. bracteatum*), revealing an intriguing evolutionary solution in some poppy species to metabolic flux and/or regulation hurdles of BIA metabolism (Farrow et al., 2015; Winzer et al., 2015).

The abstraction of hydrogen from substrates underlies P450-based catalysis as seen in the oxidation reactions discussed above. In many cases, this dehydrogenation can produce more than one radical, allowing diradical coupling, and consequently, ring formation. This structural transformation can fundamentally alter the core scaffold of the compound. Some of the earliest demonstrated examples of such P450-catalyzed C–C and C–O coupling were observed in BIA biosynthesis (Zenk et al., 1989). CYP80A1 from barberry (*Berberis stolonifera*) was the first P450 identified to catalyze a C–O coupling reaction, condensing two methylcoclaurine molecules with different enantiomeric conformations to yield the (*R*,*S*)-dimer berbamunine (Figure 1). Interestingly, although CYP80A1 is regio-specific, it can accept two (*R*)-methylcoclaurine to form the (*R*,*R*)-dimer product guattegaumerine (Kraus and Kutchan, 1995).

The CYP719A subfamily members found in isoquinoline alkaloids-producing plants are responsible for forming the methylenedioxy bridge in these compounds. In meadow rue (*Thalictrum tuberosum*), Rueffer and Zenk (1994) first observed the P450-based conversion of (*S*)-tetrahydrocolumbamine to (*S*)-canadine, also known as (*S*)-tetrahydroberberine, the precursor for many important BIAs such as noscapine, berberine, and sanguinarine. Other (*S*)-canadine synthases were later identified in several species, including CYP719A1 in Japanese goldthread (*Coptis japonica*; Ikezawa et al., 2003) and CYP719A21 in opium poppy (Dang and Facchini, 2014a). The methylenedioxy bridge formation on (*R*,*S*)-cheilanthifoline leading to (*S*)-stylopine is catalyzed by other members of this subfamily, including CYP719A2 in California poppy (Ikezawa et al., 2007) or CYP719A13 in Mexican prickly poppy (*Argemone mexicana*; Díaz Chávez et al., 2011). Ikezawa et al. (2007) also identified CYP719A3, which can accept three substrates (*R*,*S*)-cheilanthifoline, (*S*)-scoulerine, and (*S*)-tetrahydrocolumbamine to yield (*S*)-stylopine, (*S*)-nandinine, and (*S*)-canadine, respectively (Figure 1). (*S*)-Scoulerine is also subject to another methoxyphenol cyclization catalyzed by CYP719A5 in California poppy (Ikezawa et al., 2009), CYP719A14 in Mexican prickly poppy (Díaz Chávez et al., 2011), or CYP719A25 in opium poppy (Desgagné-Penix et al., 2010; Dang and Facchini, 2014b) to form (*S*)-cheinlanthifoline. Recently, CYP719A37 in black pepper (*Piper nigrum*) has been found to be responsible for the presence of the methylenedioxy bridge in piperic acid, a precursor of the pungent alkaloid piperine (Schnabel et al., 2021).

**Figure 4:**
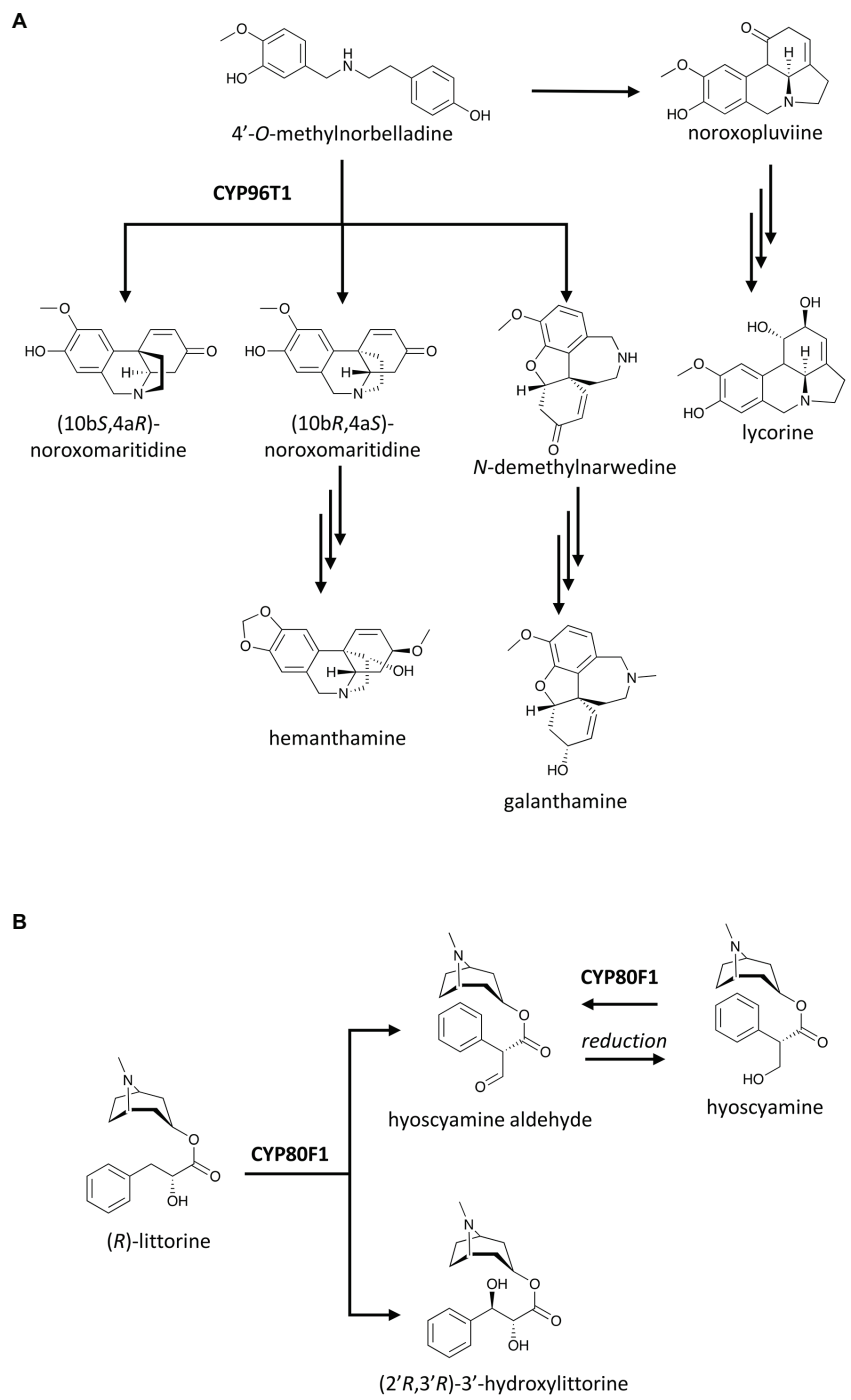
Examples of P450 catalysis beyond oxygenation including: **(A)** C–C couplings of 4'-*O*-methylnorbelladine, a central precursor of Amaryllidaceae alkaloids, leading to various pathways; and **(B)** group migration in tropane alkaloid biosynthesis. Enzymes indicated here are discussed in the text.

More than a decade after the captivating discovery of the P450-based C–O coupling reaction, the C–C coupling activity by a P450, CYP80G2, was identified in the intramolecular phenol coupling of (*S*)-reticuline that produces (*S*)-corytuberine (Ikezawa et al., 2008; Figure 1). Not too long after, another P450, CYP719B1, was found to catalyze a similar reaction on (*R*)-reticuline to form salutaridine (Gesell et al., 2009). P450-catalyzed C–C coupling also plays a significant role in the chemical diversification of Amaryllidaceae alkaloids, a group of approximately 600 isoquinoline alkaloids. As the name suggests, these alkaloids are tightly associated with the daffodil family (Amaryllidaceae) and are derived from the phenethylamine norbelladine and its derivative 4'-*O*-methylnorbelladine (Desgagné-Penix, 2021). The intramolecular C–C coupling of 4'-*O*-methylnorbelladine can occurs at different positions and stereochemistry. The *para*-*para* cyclization yields both (10b*S*,4a*R*)-noroxomaritidine and its enantiomer (10b*R*,4a*S*)-noroxomaritidine, which is the precursor for the biosynthesis of hemanthamine, pancrastatine, montanine and other *para*-*para* cyclized derivatives. The *para-ortho* cyclization affords *N*-demethylnarwedine (nornarwedine), leading to galanthamine, chlidanthine, lycoramine and similar compounds. The *ortho*-*para* coupling forms noroxopluviine, precursor of hippeastrin, lycorine and derivatives. In *Narcissus* sp. *aff. Pseudonarcissus*, CYP96T1 was identified as the enzyme that catalyzes the *para*–*para* coupling of 4'-*O*-methylnorbelladine to produce two noroxomaritidine enantiomers. This enzyme also displayed some *para*–*ortho* coupling activity as it yields *N*-demethylnarwedine as a minor product (Kilgore et al., 2016; Figure 4A). Despite this structural diversity and a long history of Amaryllidaceae plants being used in traditional medicine, galanthamine has been the only Amaryllidaceae alkaloid commercialized as a drug to treat neurodegenerative disorders. Increasing plant genomics data of Amaryllidaceae plants will undoubtedly reveal more P450s with C–C and C–O coupling activities and help us explore their untapped therapeutic potentials in the near future.

## Oxidative Ring Opening

**Figure 5:**
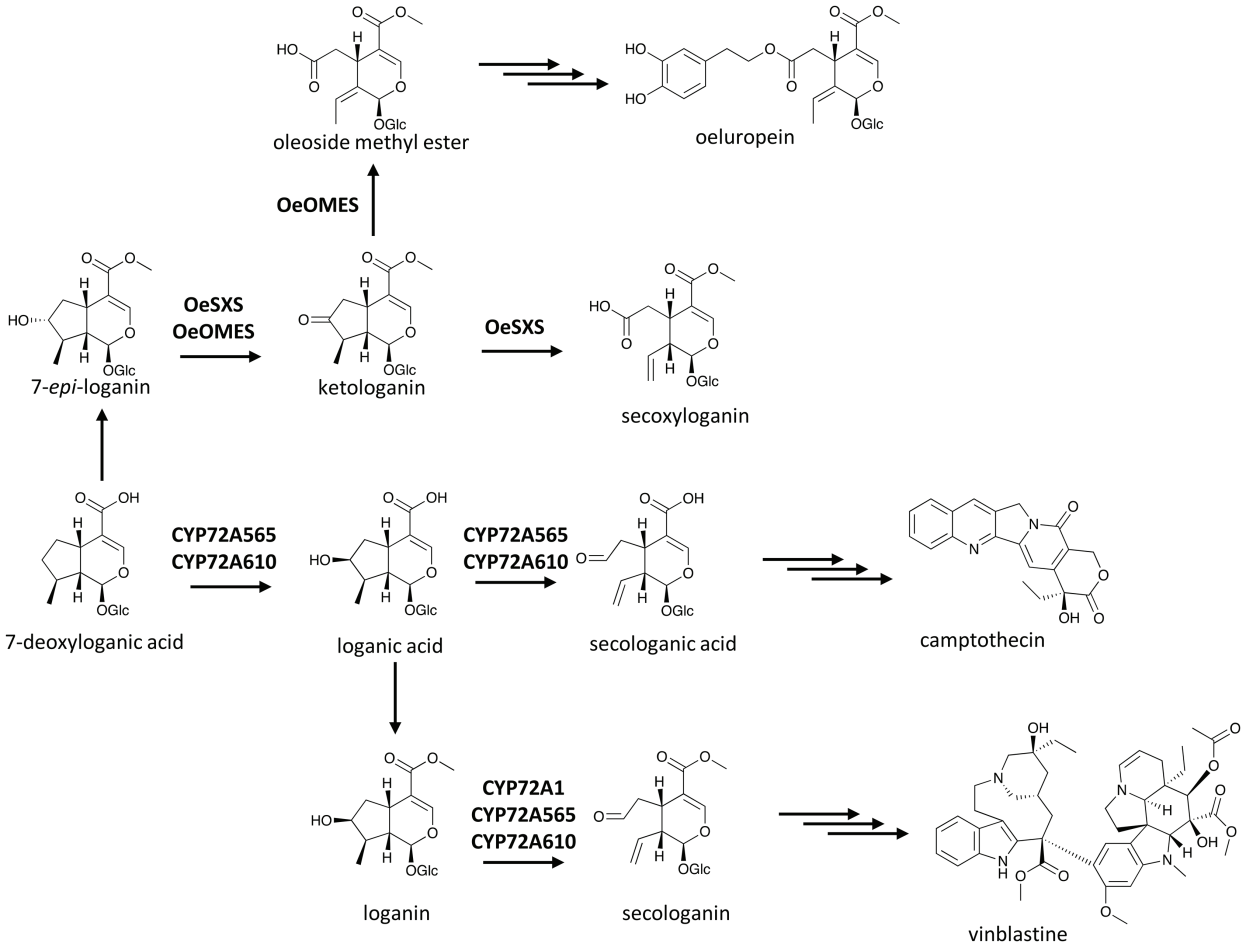
P450s in the terpenoid scaffold formation of MIAs. Multiple and single arrows indicate multiple- and single-step pathways, respectively. Enzymes indicated here are discussed in the text.

P450s are involved not only in ring formation but also in ring breakage, allowing for the formation of new scaffolds, or activate the chemicals for further condensation in alkaloid metabolism. Ring opening induced by oxygenations such as those catalyzed by CYP82X1 and CYP82N4 in BIA biosynthesis has been described earlier (Dang et al., 2015). Other P450-catalyzed ring openings leading to characteristic scaffolds have also been found. One of the earliest P450s catalyzing such unique reactions is secologanin synthase from *C. roseus*, CYP72A1, which opens the cyclopentane ring of loganin to yield secologanin (Figure 5; Irmler et al., 2000).

Secologanin synthase was the first enzyme of the then newly-found CYP72 family (Vetter et al., 1992; Irmler et al., 2000), including many members that catalyze the usual P450-based oxygenation in terpenoid metabolism (Turk et al., 2003; Ikezawa et al., 2011; Fukushima et al., 2013). CYP72A1’s unique ring opening activity is shared with close homologues in the biosynthesis of iridoids, a group of non-canonical terpenoids such as secologanin, which is condensed with tryptamine to make the central MIA precursor strictosidine (Mizukami et al., 1979; McCoy et al., 2006). In the Chinese happy tree (*Camptotheca acuminata*), both CYP72A565 and CYP72A610 display secologanin synthase activity and break the cyclopentane ring of loganic acid to produce secologanic acid, the hypothetical precursor of the anti-cancer agent camptothecin. Intriguingly, these two enzymes also hydroxylate 7-deoxyloganic acid to yield loganic acid before proceeding to open its C7–C8 bond (Figure 5; Yang et al., 2019).

Rodríguez-López et al. (2021) found two bifunctional CYP72 enzymes in olive (*Olea europaea*) with dehydrogenase activity on the hydroxyl group of 7-*epi*-loganin and C7–C8 bond breaking activity on the resulted ketologanin (Figure 5). These two enzymes, named secoxyloganin synthase and oleoside methyl ester synthase based on their products, showed that ring opening activity is not restricted to the CYP72A subfamily as they share less than 50% amino acid identity with the three CYP72A enzymes in *C. roseus* and *C. acuminata* (Irmler et al., 2000; Yang et al., 2019).

## Other Structural Re-Arrangements

The skeletal re-arrangement of (*R*)-littorine to (*S*)-hyoscyamine, a tropane alkaloid drug used to manage spasms and symptoms of Parkinson’s disease, had intrigued researchers for a long time and was hypothesized to be underlined by a P450 (Robins et al., 1995). Li et al. (2006) demonstrated that this unique migration of the whole 8-methyl-8-azabicyclo[3.2.1]octan-3-yl ester group, or re-arrangement of the 3-phenyllactate to tropate, of (*R*)-littorine is catalyzed by CYP80F1 to produce (*S*)-hyoscyamine aldehyde in black henbane (*Hyocyamus niger*). (*S*)-hyoscyamine is thought to be the precursor of (*S*)-hyoscyamine in the plant, but CYP80F1 can also oxidize the 3'-hydroxyl group of (*S*)-hyoscyamine back to (*S*)-hyoscyamine aldehyde. In addition, CYP80F1 displays remarkable catalytic promiscuity by catalyzing a single oxidation without group migration at C3' position of (*R*)-litorine to yield (2'*R*,3'*R*)-3'-hydroxylittorine (Figure 4B; Li et al., 2006).

Colchicine biosynthesis again showcases the fascinating diversity of P450 activities. In addition to the two CYP75A enzymes described earlier, the metabolic pathway of colchicine involves a unique P450 with ring expansion activity. This P450-based ring expansion was demonstrated using NADPH and microsome from the seed of autumn crocus (*Colchicum autumnale*) by Rueffer and Zenk (1998), and the enzyme was identified more than 20 years later by Nett et al. (2020) as CYP71FB1. In this reaction, the 1,4-cyclohexadiene ring of *O*-methylandrocymbine is expanded to yield *N*-formyldemecolcine, the precursor of colchicine. Although the final steps leading to colchicine are yet to be elucidated, the finding of CYP71BF1 activity has established how the characteristic tropolone ring of colchicine structure is built (Figure 3).

## General Discussion

P450 enzyme control occurs at many steps in all chemical diversification pathways of alkaloids. The catalytic versatility of P450 enzymes provides not only tremendous chemical diversity and thus adaptability to increase plants’ fitness but also blueprints for biocatalyst engineering with applications in medicine, industry, and bioremediation (Bernhardt, 2006; Nelson and Werck-Reichhart, 2011; Sakaki, 2012; Li et al., 2020; Shang and Huang, 2020).

Although enzymes in the same P450 family tend to catalyze similar reactions in alkaloid metabolism, such as single hydroxylation by CYP82 members and methylenedioxy bridge formation by CYP719 enzymes, this is not always the case. Increasingly abundant genomic data will allow for the identification of more P450s and their roles in alkaloid biosynthesis in natural products biochemistry in general (Nelson, 2018). Given the complexity of alkaloid structures and the sheer number of unelucidated pathways, what appear to be “unusual” such as the ring-expanding functions by CYP71BF1 or a P450-dependent oxidoreductase fusion like CYP82Y2 could turn out to occur in other pathways and provide excellent templates for future enzyme engineering to harness these unique chemical prowesses. Likewise, understanding differential substrate specificities of P450s acting in the same pathway and on similar substrates as observed in the noscapine pathway sheds light on the chemical strategies that plants employ and thus inform future pathway engineering approaches. Recent progress in exploring plant P450s have afforded the production in engineered hosts of alkaloids such as dihydrosanguinarine, noscapine (Li et al., 2018), thebaine, hydrocodone (Thodey et al., 2014), strictosidine (Brown et al., 2015), vindoline (Qu et al., 2015), and *N*-formyldemecolcine (Nett et al., 2020).

The broad substrate spectrum of many P450s can complicate biosynthesis studies and metabolic engineering efforts (Hidalgo et al., 2017; De La Peña and Sattely, 2021). Nevertheless, such promiscuity sheds light into the evolution of these enzymes, and indicates their crucial role as part of the catalytic reservoirs whose members can be recruited for emerging pathways and further drive the chemical diversity of plants (Tawfik, 2010; Ikezawa et al., 2011; Weng et al., 2012; Guo et al., 2016; Dang et al., 2017, 2018; Forman et al., 2018; Christ et al., 2019; Nguyen et al., 2019; Lichman et al., 2020; Nguyen and O’Connor, 2020). Even for non-native or new-to-nature substrates including halogenated analogues, P450s display a certain degree of natural tolerance as observed in the multiple-step biotransformation of 7-chlorotryptamine to 12-chloro-19,20-dihydroakuammicine in MIA metabolism of *C. roseus* cultures (Glenn et al., 2011). This feature may, therefore, also provide natural templates for catalytic optimization towards desired and/or novel yet related activities. Despite the general challenge due to the lack of P450 structural information and the requirement of redox partners, P450 engineering will undoubtedly benefit from the cataloguing of new sequences and functions from the ever-expanding plant genome datasets. In addition, modification of the relatively-conserved substrate-recognition sites across P450s may allow product profile customization without experimental protein structural data (Gotoh, 1992; Forman et al., 2018; Shang and Huang, 2020).

There remain other challenges in understanding the catalytic mechanisms of P450 enzymes beyond substrate specificities such as non-oxidative reactions, while the membrane-bound nature of plant P450 enzymes impedes structural studies using crystallography approaches (Shang and Huang, 2020; Zhang et al., 2020). In addition, the interaction and localization of P450 enzymes with respect to other enzymes in the same pathways remain to be explored (Bassard et al., 2017). All of these continue to fascinate scientists for the years to come.

## Author Contributions

TTTD and TDN wrote the manuscript together. All authors contributed to the article and approved the submitted version.

### Conflict of Interest

The authors declare that the research was conducted in the absence of any commercial or financial relationships that could be construed as a potential conflict of interest.
